# Presidential Address

**Published:** 1902-08-02

**Authors:** Walter Whitehead

**Affiliations:** President of the Association; Consulting Surgeon to the Royal Infirmary, Manchester.


### The British Medical Association.

### PRESIDENTIAL ADDRESS.

By Walter Whitehead, F.R.C.S.Edin., F.R.S.Edin., President of the Association; Consulting Surgeon to the Royal Infirmary, Manchester.

### Manchester's Early Influence on the Advancement of Medicine and Medical Education.

Mr. Whitehead began by saying that his first duty was to thank his fellow members of the British Medical Association who had chosen him to fill the honourable office of President, and had thus afforded him the opportunity of welcoming them to the city of Manchester at this, the seventieth annual meeting of the Association, the largest voluntary medical society in the world. In extending to all our visitors a hearty welcome, I speak, he said, not only in the name of the members of the Association resident in Manchester and the district around, but also of the citizens of Manchester, who are represented here to-night by their chief magistrate, the Right Hon. the Lord Mayor, and who join with us in hoping that your visit to our city may be both useful and pleasant.

When the British Medical Association last held its annual meeting in Manchester, just twenty-five years ago, the medical school had only recently been amalgamated with the Owens College, of which it has ever since been one of the most important departments. The College, which celebrated its jubilee a few years ago, owes its success as much to the breadth of view and wise foresight of its founder as to his noble generosity. This day, July 29th, is the fifty-sixth anniversary of the death of John Owens, and I ask you to join with me in praising the memory of one who was among the first in this country to show an appreciation of the fact, now so widely recognised, that the prosperity of a community, as of a nation, must have for one of its foundation stones a true respect for learning and intellectual inquiry, and an ardent desire to place the advantages of education within the reach of all who are capable of profiting by them.

After an eloquent tribute to the memory of the many well-known men who took an active part in the organisation of the annual meeting in Manchester in 1877 who have since passed away, Mr. Whitehead gave a short outline of the many matters of interest, in addition to the proper work of the meeting, to which the visitors to Manchester might profitably and pleasantly turn their attention.

He then turned to his main theme, in the course of which he gave an admirable historical review of the development of, and especially the early development of, those medical, scientific, educational, and charitable institutions which have made Manchester famous all the world over in quite a different sense from that suggested by its commercial importance.

In conclusion, he said that from a very early date in the history of the British Medical Association the medical profession in and around Manchester had taken a deep interest in its welfare and development. The British Medical Association was founded in 1831, and in 1835 the fourth anniversary meeting was held in Manchester under the presidency of Dr. Edward Holme, F.R.S., who had long held the office of physician to the Royal Infirmary. This meeting, the first held in Manchester, was memorable in the history of the Association as that at which the system of Branch organisation took origin by the amalgamation with the parent association of the Eastern Provincial Medical and Surgical Association, which became the East Anglian Branch. Manchester thus saw the first great extension of the local organisation of the British Medical Association, and this year it sees another great step towards the perfecting of that organisation. The Association held its annual meeting again in Manchester in 1854, when Mr. William Wilson, surgeon to the Royal Infirmary, and well known for his important contributions to obstetrics and gynæcology, presided. The membership had grown to over 2,000, and an important proposal, marking a further stage in the development of the Association, was made. This was that the title should be changed from the Provincial Medical and Surgical Association, which it had hitherto borne, to that of the British Medical and Surgical Association. This change of name betokened an extension of the Association's work to embrace all British practitioners of medicine. Finally, after a good deal of discussion, the familiar title of the British Medical Association was chosen, and formally adopted in 1856. Under that name it had grown and flourished, and it would, he hoped and believed, continue to flourish, serving as a connecting link between the members of their scattered profession—scattered in their student days in many schools, obtaining their diplomas and degrees from many universities and corporations, and ultimately practising in every district of the United Kingdom and every region of a united empire.

